# Acute kidney injury in autologous hematopoietic stem cell transplant for patients with lymphoma – KDIGO classification with creatinine and urinary output criteria: a cohort analysis

**DOI:** 10.1080/0886022X.2023.2183044

**Published:** 2023-03-01

**Authors:** Natacha Rodrigues, Carolina Branco, Claúdia Costa, Filipe Marques, Marta Neves, Pedro Vasconcelos, Carlos Martins, José António Lopes

**Affiliations:** a Division of Nephrology and Renal Transplantation, Centro Hospitalar Universitário Lisboa Norte, EPE, Lisboa, Portugal; b Division of Hematology, Centro Hospitalar Universitário Lisboa Norte, EPE, Lisboa, Portugal

**Keywords:** Acute kidney injury, hematopoetic stem cell transplant, lymphoma, epidemiology

## Abstract

Eligibility and indication for autologous hematopoietic stem cell transplantation (HSCT) in patients with lymphoma are increasing. Acute kidney injury (AKI) is a known complication of HSCT with studies including a miscellaneous of hematological diagnoses and using different definitions of AKI. We aimed to evaluate incidence, risk factors and prognostic impact of AKI post-HSCT in patients with lymphoma submitted to autologous HSCT using the KDIGO classification with both serum creatinine and urinary output criteria. We performed a single-center retrospective cohort study including patients with lymphoma admitted for autologous HSCT. We used survival analysis with competing risks to evaluate cumulative incidence of AKI, AKI risk factors and AKI impact on disease-free survival. We used Cox regression for impact of AKI on overall survival. We used backward stepwise regression to create multivariable models. A total of 115 patients were included. Cumulative incidence of AKI: 63.7% 100 d post-HSCT. First diagnosis criteria: creatinine in 54.8%, urinary output in 41.1% and both in 4.1%. AKI highest stage: 1 in 57.5%, 2 in 17.8% and 3 in 24.7%. Variables independently associated with higher incidence of AKI were: use of nephrotoxic drugs (HR: 2.87, 95% CI: 1.07–7.65; *p* = 0.035), mucositis (HR: 1.95, 95% CI: 1.16–3.29; *p* = 0.012) and shock (HR: 2.63, 95% CI: 1.19–5.85; *p* = 0.017). Moderate to severe AKI was independently associated with lower overall survival (HR: 2.04, 95% CI: 1.06–3.94; *p* = 0.033). No association with relapse nor progression to chronic kidney disease (CKD) was found. AKI affects almost two thirds of patients with lymphomas submitted to autologous HSCT. Nephrotoxic drugs, mucositis and shock are important independent AKI risk factors. More than one third of AKI episodes are moderate to severe and these are associated with lower overall survival.

## Introduction

Over the last decades, we have witnessed an increase in the incidence of hematological malignancies and the development of new chemotherapy induction regimens. Hematopoietic stem cell transplantation (HSCT) has thus become an important therapeutic option for hemato-oncologic patients. Being consensually associated with significantly better survival rates in eligible patients, autologous HSCT is the most frequent and intensive approach for the treatment of lymphoma patients [1]. With a growing incidence of autologous HSCT in patients with lymphoma comes the necessity of better understanding complications and related clinical implications. Acute kidney injury (AKI) is an important complication in the first 100 d post HSCT in general, with studies showing an incidence ranging from 20 to 92% and a negative impact on overall survival [2–6]. These studies tend to include several hematological diagnoses submitted to different types of HSCT, as well as heterogeneous definitions for AKI not following more recent classifications and not accounting for urinary output.

AKI in autologous HSCT has been less studied than in allogeneic HSCT and include not only patients with lymphoma but also patients with multiple myeloma and/or amyloidosis, which have recognized different clinical characteristics and prognosis and may, in turn, result in less accurate data when extrapolating the results for each hematological diagnosis.

For the definition of AKI, the most updated and consensual classification is the KDIGO classification [7]. It was proposed in 2012 and resulted from the fusion of the former classifications Risk Injury Failure Loss of kidney function End-stage kidney disease – RIFLE in 2004, and Acute Kidney Injury Network – AKIN in 2007 [8,9] (RIFLE and AKIN) to establish one classification of AKI for clinical practice, research and public health. To its full extent, it takes in consideration both serum creatinine elevation and urinary output reduction. Few recent studies on AKI in HSCT in general used the KDIGO classification and, even those, used only the creatinine elevation criteria for the KDIGO classification.

All these aspects enlighten the need for studies focusing on AKI in patients with lymphoma submitted to autologous HSCT, considering AKI definition by KDIGO whilst using both serum creatinine elevation and urinary output reduction.

Our study aims to 1) determine incidence and severity of AKI in patients with lymphoma submitted to autologous HSCT using KDIGO classification with both creatinine and urinary output criteria, 2) to identify independent risk factors for AKI in these patients and 3) to evaluate the impact of AKI on disease-free survival, on overall survival in the first 3 years after HSCT 4) To evaluate progression to chronic kidney disease (CKD**)** and estimated glomerular filtration rate (eGFR) reduction superior to 25% at the end of the first year and at the end of the 3 years after HSCT.

## Materials and methods

### Study design, population, and data collection

We performed a single-center retrospective cohort study. Our study population included patients who were diagnosed with lymphoma and submitted to autologous HSCT at the Centro Hospitalar Universitário Lisboa Norte, EPE (CHULN) between January 2005 and December 2015. As exclusion criteria we defined: Patients under the age of 18 years, patients with CKD already on renal replacement therapy; patients who underwent renal replacement therapy one week before transplantation and those with previous HSCT.

The conditioning regimens used followed institutional protocols – Carmustine, Etoposide, Cytarabine and Melphalan (BEAM) or Thiotepa, Etoposide, Cytarabine and Melphalan (TEAM). Total body irradiation is not available in our institution, and it is not contemplated in any of our institutional protocols.

Our data collection was based on registers of daily medical records, 6-h period nurses’ records and diagnostic exams during hospital admission period for HSCT, as well as all routine medical records and laboratorial analysis before and after HSCT. We collected variables related to patient demographic characteristics (age, gender, race, body weight, and height), related to patient comorbidities (diabetes mellitus, hypertension, arrythmia, valvular heart disease, ischemic heart disease, cerebrovascular disease, chronic liver disease, intestinal inflammatory disease, peptic ulcer, connective tissue disease, chronic obstructive pulmonary disease, and solid-organ cancer, psychiatric disease), related to lymphoma and previous treatment approach (subtype of lymphoma, number of previous lines of therapy, and exposure to radiotherapy in the past); related to HSCT (conditioning regimen, cells source, period of aplasia, length of stay in hospital, blood results on hospital admission day for HSCT, sinusoidal obstructive syndrome, thrombotic microangiopathy, sepsis, nephrotoxic drugs, shock, cytomegalovirus infection, AKI, and AKI stage) and related to prognostic impact (time to relapse, time to all-cause mortality and eGFR 1 year after HSCT and 3 years after HSCT).

All patients were followed until death or censored at 36 months (3 years) after HSCT. This timeline was defined because patients are often transferred to other hospitals closer to their residence for continued follow-up after this 3-year period.

### Definitions

We considered serum creatinine at the last medical appointment before hospital admission for HSCT to be baseline serum creatinine. Baseline glomerular filtration rate was estimated according to CKD-EPI equation [10], using baseline serum creatinine.

AKI diagnosis was made based on daily values of serum creatinine and 6-h urinary output since the time of hospital admission for HSCT until hospital discharge, as well as all other hospital admissions or weekly evaluation in outpatient clinic in the first 100 d after HSCT. AKI was defined by KDIGO criteria [7] (any of the following: Increase in serum creatinine by ≥0.3 mg/dl (≥26.5 µmol/l) within 48 h; or increase in serum creatinine to ≥1.5 times baseline, which is known or presumed to have occurred within the prior 7 d; or urinary output <0.5 ml/kg/h for 6 h). Stage of AKI followed KDIGO classification considering the worst serum creatinine value and/or longest period of urinary volume reduction during hospital stay for HSCT. Moderate to severe AKI was defined as AKI stage 2 and AKI stage 3.

CKD was defined as a persistent decrease in eGFR to below 60 ml/min/1.73 m^2^, according to the definition of KDIGO [11].

The hematopoietic cell transplantation – specific comorbidity index (HCT-CI) [12] was calculated according to the latest validated version considering patients comorbidities. Shock was considered when patients presented with cardiac frequency >90 bpm, systolic blood pressure <90 mmHg, and at least one lactate determination >2 mmol/l or 22 mg/dl.

Nephrotoxic drugs included gentamicin, amikacin, vancomycin, amphotericin B, and foscarnet.

### Ethical committee

This study was approved by the local Ethical Committee (approval number 35336) in agreement with institutional guidelines. Informed consent was waived by the Ethical Committee due to the retrospective and non-interventional nature of the study.

### Statistical methods

Categorical variables were described as frequencies, continuous data were expressed as median and interquartile range (P25 = 25th percentile; P75 = 75th percentile).

We followed the statistical methodology suggested by the European Group for Blood and Marrow Transplantation [13]. We used survival analysis methods considering competing events – we considered death as a competing risk event – through the Fine and Gray method [14] to calculate cumulative incidence of AKI. We also used this survival analysis method to perform univariable and multivariable analyses of factors predicting AKI. We used backward stepwise regression to create the final multivariable model. This same strategy was applied to calculate the impact of AKI on disease-free survival. AKI impact on overall survival was evaluated using cox regression model followed by backward stepwise regression to create the final multivariable model. We considered type 1 right censoring for a period of three years after HSCT. We calculated the incidence of CKD and eGFR reduction >25% in survivors 1 year after HSCT and survivors 3 years after HSCT and evaluated association with AKI in the first 100 d post HSCT.

Analyses were performed with the statistical software package STATA version 16.0 for Windows (StataCorp, College Station, TX).

## Results

One hundred and forty-three patients with lymphoma were submitted to autologous HSCT at our center between January 2005 and December 2015. Among these patients, 28 patients were excluded for presenting at least one exclusion criteria and 115 patients were eligible for the study. Patients’ baseline characteristics and transplant-related aspects are shown in Table 1.

**Table 1.** Patients’ baseline characteristics and transplant-related variables.

Patients characteristics	Category	*n* (%)	P50	P25	P75
Age at transplant (years)			50.2	33.9	59.5
Gender	Male	59 (51.3)			
Race	Caucasian	105 (91.3)			
BMI (kg/m^2^)			25.3	21.8	35.9
HCT-CI	0–1	97 (84.4)			
	≥2	18 (15.6)			
Hematologic diagnosis	B-cell lymphomas	73 (63.5)			
	Hodgkin lymphomas	37 (32.2)			
	T-cell lymphomas	5 (4.4)			
Nr of prior cycles of therapy			9	7	10
Previous radiotherapy	yes	22 (19.3)			
Basal eGFR (ml/min/1.73 m^2^)			107.5	94.3	124.6
Induction regimen	BEAM	109 (94.8)			
	TEAM	6 (5.2)			
Cells source	perihperal blood	111 (96.5)			
	bone marrow	4 (3.5)			
Period of aplasia (days)			10	10	11
Sepsis		26 (22.6)			
Nephrotoxic drugs		103 (89.6)			
Mucositis		74 (64.4)			
TMA/TLS/SOS		6 (5.3)			
Shock		8 (6.9)			
At hospital admission day:					
Hemoglobin (gr/dl)			11.5	10.3	12.7
Leukocytes (cells/mm^3^)			5620	4000	7340
Neutrophils (cells/mm^3^)			3530	2490	5290
Lymphocytes (cells/mm^3^)			1040	800	1820
Platelets (/μl)			194,000	140,000	273,000
Urea (mg/dl)			34	28	39
Uric Acid (mg/dl)			5	4.2	6
Calcium (mg/dl)			9	8.9	10
Phosphate (mg/dl)			4	3.2	4.1
Reactive C protein (mg/dl)			0.52	0.21	1
Lactate dehydrogenase (U/l)			342	297	419
Albumin (mg/dl)			4.1	3.8	4.4
Alanine transaminase (U/l)			21	15	30
Total bilirubin (mg/dl)			0.4	0.3	0.6

P50: median; P25–25th percentile: P75–75th percentile; P25 BMI: body mass index; HCT-CI: hematopoietic stem cell transplant comorbidity index; Nr: number; eGFR: estimated glomerular filtration rate; BEAM: carmustine, etoposide, cytarabine, and melphalan; TEAM: thiotepa, etoposide, cytarabine, and melphalan; TMA/TLS/SOS: thrombotic microangiopathy, tumor lysis syndrome, or sinusoidal obstruction syndrome.

### AKI cumulative incidence, presentation, and severity

**Figure 1.**
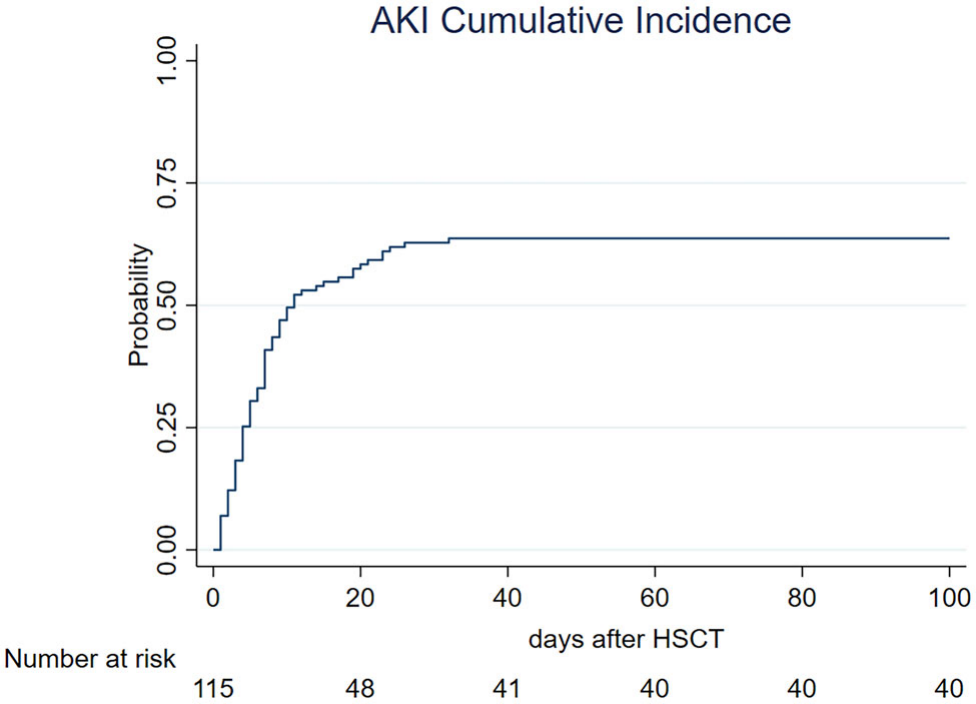
Cumulative incidence of AKI post HSCT. Cumulative incidence function of AKI according to the KDIGO classification using serum creatinine rise criteria and urinary output criteria. Death was considered a competing event. AKI: acute kidney injury.

The AKI cumulative incidence was 63.7% at 100 d after autologous HSCT (Figure 1).

The moderate-to-severe AKI cumulative incidence was 27.1% at 100 d after autologous HSCT.

The earlier diagnosis criteria for AKI were serum creatinine rise in 54.8% of patients and urinary output reduction in 41.1% of patients. Both serum creatinine rise and urinary output reduction were observed in the first day of AKI in 4.1% of patients.

In the first day of AKI, patients presented the following severity: AKI Stage 1 = 80.8%, AKI Stage 2 = 12.3%, and AKI Stage 3 = 6.9% (moderate-to-severe AKI in 19.2%). As AKI occurred, the highest severity registered was AKI Stage 1 = 57.5%, AKI Stage 2 = 17.8%, and AKI stage 3 = 24.7% (moderate to severe AKI in 42.5%).

### AKI risk factors

In the univariable analysis, the predictor variables with impact on AKI were HCT-CI score ≥2 (HR: 1.74, 95% CI: 1.04–2.90; *p* = 0.034), use of nephrotoxic drugs (HR: 3.12, 95% CI:1.18–8.20; *p* = 0.021), mucositis (HR:2.13, 95% CI: 1.28–3.52; *p* = 0.003), thrombotic microangiopathy, or sinusoidal obstructive syndrome ((HR: 2.52, 95% CI: 1.41–4.50; *p* = 0.002), shock (HR: 4.11, 95% CI: 2.28–7.45; *p* = < 0.001) (Table 2).

**Table 2.** Competitive risk regression. Univariable analysis for AKI.

Patients and transplant-related characteristics	Hazard ratio estimate	95% Confidence interval		*p* Value
		Lower limit	Upper limit	
Age at transplant (years)	1	0.99	1.02	0.393
Gender	0.84	0.54	1.31	0.457
Race	0.94	0.38	2.35	0.903
BMI (kg/m^2^)	1.03	0.99	1.08	0.185
HCT-CI ≥ 2	1.74	1.04	2.9	0.034
Hematologic diagnosis:				
Hodgkin lymphomas comparing B-cell lymphomas	1.1	0.67	1.81	0.69
B-cell lymphomas comparing T-cell lymphomas	1.21	0.53	2.8	0.648
Hodgkin lymphomas comparing T-cell lymphomas	1.33	0.56	3.18	0.532
Nr of prior cycles of therapy	0.98	0.91	1.05	0.647
Previous radiotherapy	1	0.57	1.77	0.977
basal eGFR (ml/min/1.73 m^2^)	0.99	0.98	1	0.192
Induction regimen	0.63	0.22	1.82	0.398
Cells source	1.74	0.55	5.55	0.348
Period of aplasia (days)	0.95	0.82	1.11	0.54
Sepsis	0.8	0.5	1.26	0.347
Nephrotoxic drugs	3.12	1.18	8.2	0.021
Mucositis	2.13	1.28	3.52	0.003
TMA/SOS	2.52	1.41	4.5	0.002
Shock	4.11	2.28	7.45	<0.001
At hospital admission day:				
Hemoglobin (gr/dl)	1.01	0.87	1.16	0.908
Leukocytes (cells/mm^3^)	1	1	1	0.447
Neutrophils (cells/mm^3^)	1	0.99	1	0.672
Lymphocytes (cells/mm^3^)	1	0.99	1	0.523
Platelets (/μl)	1	0.99	1	0.603
Urea (mg/dl)	1.01	0.99	1.01	0.172
Uric Acid (mg/dl)	1.06	0.94	1.21	0.326
Calcium (mg/dl)	0.88	0.56	1.4	0.586
Phosphate (mg/dl)	0.92	0.7	1.2	0.523
Reactive C protein (mg/dl)	0.96	0.88	1.04	0.312
Lactate dehydrogenase (U/l)	1	1	1	0.292
Albumin (mg/dl)	0.94	0.59	1.52	0.812
Alanine transaminase (U/l)	1	0.99	1.01	0.333
Total bilirubin (mg/dl)	0.92	0.76	1.14	0.481

BMI: body mass index; HCT-CI: hematopoietic stem cell transplant comorbidity index; Nr; number; eGFR: estimated glomerular filtration rate; BEAM: carmustine, etoposide, cytarabine, and melphalan; TEAM: thiotepa, etoposide, cytarabine, and melphalan; TMA/TLS/SOS: thrombotic microangiopathy, tumor lysis syndrome, or sinusoidal obstruction syndrome.

In the multivariable analysis, variables independently associated with a higher incidence of AKI included: nephrotoxic drugs (HR: 2.87, 95%CI:1.07–7.65; *p* = 0.035), mucositis (HR:1.95, 95% CI:1.16–3.29; *p* = 0.012), and shock (HR: 2.63, 95% CI:1.19–5.85; *p* = 0.017) (Table 3).

**Table 3.** Competitive risk regression. Multivariable analysis for AKI.

Patients and transplant-related Characteristics	Hazard ratio estimate	95% Confidence interval		*p* Value
		Lower limit	Upper limit	
Nephrotoxic drugs	2.87	1.08	7.65	0.035
Mucositis	1.95	1.16	3.29	0.012
MAT/TLS/SOS	2.00	0.73	5.54	0.182
Shock	2.64	1.19	5.85	0.017
Gender	0.97	0.61	1.52	0.882
BMI	1.02	0.97	1.08	0.428
HCT-CI ≥ 2	1.48	0.81	2.69	0.200
Age at transplant	0.99	0.97	1.01	0.378
basal eGFR	0.99	0.98	1.00	0.125
Cells source	1.71	0.66	4.44	0.273
Induction regimen	0.69	0.22	2.16	0.523

BMI: body mass index; HCT-CI: hematopoietic stem cell transplant comorbidity index; eGFR: estimated glomerular filtration rate; TMA/TLS/SOS: thrombotic microangiopathy, tumor lysis syndrome, or sinusoidal obstruction syndrome.

### AKI prognostic impact

At the end of the three years after autologous HSCT, 42 (36.5%) patients had died. Fifty-two percent of deaths occurred within the first year after autologous HSCT.

In univariable analysis, the variables with impact on mortality were relapse (HR:6.24, 95%CI:2.94–13.24; *p*= <0.001) and moderate to severe AKI ((HR:2.04, 95%CI:1.06–3.94; *p* = 0.033).

In multivariable model, the variables that were independently associated with a lower overall survival were relapse (HR:5.34, 95%CI:2.55–22.30; *p*= <0.001), BMI (HR:0.91, 95%CI:0.84–0.91; *p*= 0.025) and moderate to severe AKI ((HR:2.05, 95%CI:1.10–3.82; *p* = 0.024) (Table 4).

**Table 4.** Cox proportional hazards model regression. Multivariable analysis for mortality.

Patients and transplant-related characteristics	Hazard ratio estimate	Standard error	95% Confidence interval		*p* Value
			Lower limit	Upper limit	
Relapse	6.24	2.39	2.94	13.23	<0.001
Moderate to severe AKI	2.04	0.68	1.06	3.94	0.033
Age at transplant	1.02	0.01	0.99	1.04	0.18
Race	3.42	3.69	0.41	28.52	0.256
BMI	0.91	0.04	0.84	0.99	0.025
HCT-CI ≥ 2	2.08	0.88	0.91	4.76	0.083

AKI: acute kidney injury; BMI: body mass index; HCT-CI: hematopoietic stem cell transplant comorbidity index.

**Figure 2.**
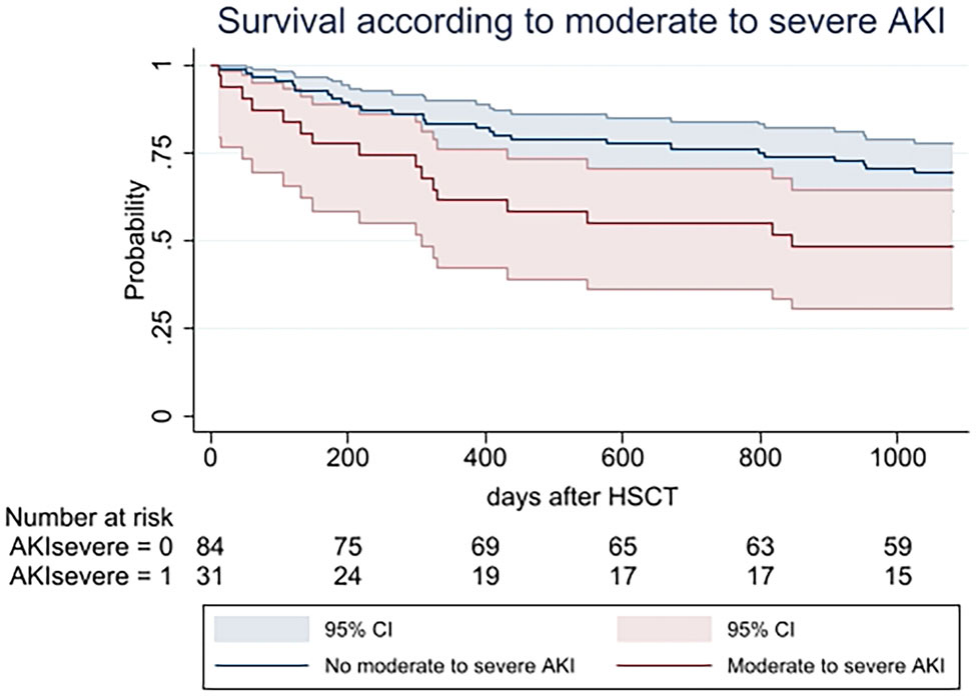
Overall survival considering moderate-to-severe AKI. Overall survival in days in the first 3 years of HSCT considering moderate to severe AKI according to the KDIGO classification using serum creatinine rise criteria and urinary output criteria. AKI: acute kidney injury; CI: confidence interval; HSCT: hematopoietic stem cell transplant.

Overall survival according to moderate-to-severe AKI is shown in Figure 2.

Cumulative incidence of relapse was 45.2% at three years after HSCT. No statistically significant association was found between AKI (nor moderate-to-severe AKI) with lower disease-free overall survival.

None of the patients had CKD previous to HSCT and their median eGFR was 107.5 (94.3–124.6) ml/min/1.73 m^2^. One year after HSCT none of the survivors had CKD and the median eGFR was 103.7 (87.9–121.6) ml/min/1.73 m. Despite the absence of CKD, 31.2% of the survivors had an eGFR reduction superior to 25% comparing to the baseline previous to HSCT. No association was found between this reduction and previous AKI (*p* = 0.730).

Three years after HSCT 2.2% of the survivors had CKD and the median eGFR was 98.6 (80.1 − 115.8) ml/min/1.73 m. An eGFR reduction superior to 25% was registered in 31.8% of the survivors when comparing to the baseline eGFR previous to HSCT and 10% when comparing to eGFR 1 year after HSCT. No association was found between this reduction and previous AKI (*p* = 0.113).

## Discussion

In our study we found a cumulative incidence of AKI and moderate-to-severe AKI of 63.7% and 27.1%, respectively, using KDIGO classification and both creatinine and urinary output criteria.

A meta-analysis published in 2020 by Kanduri et al. considering 5144 patients submitted to either allogeneic or autologous HSCT presented an incidence of AKI and severe AKI of 55.1% and 8.3% [15]. Considering autologous HSCT studies, data by Lopes et al. show an AKI incidence of 12% [16], Fadia et al. found an AKI incidence of 21% [17], Caliskan et al. found and AKI incidence of 52% [18] and, similarly, Merouani et al. report an AKI incidence of 56% [19]. These previous studies used different AKI definitions and did not take in consideration urinary output criteria. Our higher incidence compared to other studies on autologous HSCT may be explained by a more updated and complete AKI definition (KDIGO classification including both creatinine and urinary output criteria) which we believe to contribute to more accurate results.

One aspect we consider worth notice in our study is that although moderate-to-severe AKI represented 19.2% of AKI cases on AKI day of onset, this severity represented 41.5% when considering the highest stage reached. These grades of severity were independently associated with lower overall survival. An earlier approach in less severe AKI stages may reduce the probability of progression to more severe stages and improve outcomes. In our study, urinary output reduction alone was the first criteria to be present on the day of AKI onset in more than forty percent of AKI patients. Thus, urinary output monitorization is of major importance in these patients and should be taken in account in clinical practice to approach AKI as soon as possible to prevent progression of AKI.

Known risk factors and causes for AKI in the general population - namely nephrotoxic drugs and episode of shock – had an expected significantly higher incidence in patients with AKI in our lymphoma patients submitted to autologous HSCT. This result has been consistently suggested in other studies [19–23]. Focusing on other risk factors and causes of AKI specific of HSCT, mucositis showed a strong statistically significant association with AKI, which was also found by Andronesi et al. in his study on autologous HSCT in multiple myeloma patients [24].

Our study did not find an association between lower baseline eGFR with AKI as other autologous HSCT studies did. We believe this is related to the fact that our patients did not have CKD (eGFR was 107.5 ml/min/1.73 m^2^ (94.3–124.6 IQR), as patients with lymphoma tend to be much younger and previously healthier than patients with multiple myeloma or amyloidosis (included in other autologous HSCT studies).

AKI is a risk factor for lower short- and long-term overall survival in different scenarios and populations, and there is a graded relationship between severity of AKI and increased mortality [25–28]. Although our results on overall survival with a 3-year follow up period in patients with lymphoma submitted to autologous HSCT had no significant association with AKI, moderate-to-severe AKI showed an independent association with lower overall survival (HR:2.05, 95%CI:1.10–3.82; *p* = 0.024). Andronesi et al. also did not find higher mortality rates in autologous HSCT with AKI including all stages [24] and studies considering HSCT in general have demonstrated that mortality rates increase as the severity of AKI increases [29–31]. Our results enlighten this finding in autologous HSCT in lymphoma patients and reinforce the need for monitoring and an early approach to AKI in these patients to prevent progression to higher AKI stages ultimately associated with worse prognosis.

Our population did not have CKD previous to the HSCT. We registered a reduction of eGFR > 25% in around one quarter of the patients but only 2.2% of patients progressed to CKD 3 years after HSCT. These renal outcomes were not significantly associated to previous AKI although Gutierrez et al. [32] and Sakaguchi et al. [33] found an association between AKI and progression to CKD in HSCT. These authors focused on allogeneic HSCT in patients with leukemia and multiple myeloma. Allogeneic HSCT includes aggressive induction chemotherapy as well as calcineurin inhibitors chronically administered after the HSCT and patients have different outcomes related to their baseline disease. Also, these studies included patients with CKD prior to HSCT which is a known risk factor and had longer follow-up periods. Still, we believe that we would need higher number of patients considering the high mortality rate of our population and longer period of follow up to infer long-term impact on renal outcomes.

As a single-center retrospective study, we acknowledge limitations related to external validity and data availability. Patients had often two or more nephrotoxic drugs previous to AKI which does not allow to extract information on which nephrotoxic had higher impact on AKI. Also, other criteria for CKD like proteinuria or structural abnormalities were not available as well as other serum creatinine measures between 1 year and 3 years after HSCT.

Despite these limitations, to the best of our knowledge, this is the first study focusing exclusively on this hematological malignancy group submitted to autologous HSCT using KDIGO classification. It is also the first study to consider not only creatinine criteria but also UO criteria to diagnose and categorize AKI in an HSCT population.

More studies are needed in this population of patients defining AKI by KDIGO classification through both creatinine and urinary output criteria, particularly prospective and multicentric studies.
